# A Novel Indolium-Based Fluorescent Probe for Fast Detection of Cyanide

**DOI:** 10.3390/bios14050244

**Published:** 2024-05-13

**Authors:** Mei Ding, Xiao Xiao, Chen Zhou, Mingxin Luo, Jing Sun

**Affiliations:** School of Chemistry & Environmental Engineering, Jilin Provincial International Joint Research Center of Photo-Functional Materials and Chemistry, Changchun University of Science and Technology, Changchun 130022, China; dingmei@cust.edu.cn (M.D.); xiaoxiao@cust.edu.cn (X.X.); luomingxin@cust.edu.cn (M.L.); sunjing@cust.edu.cn (J.S.)

**Keywords:** cyanide, fluorescent probe, indole derivative

## Abstract

A novel indolium-based fluorescent probe for the detection of CN^−^ was developed based on the conjugation of 1, 2, 3, 3-Tetramethyl-3H-indolium iodide and 2-acetyl benzothiophene. The introduction of external CN^−^ caused a nucleophilic attack to the quaternary amine salt structure in the probe and resulted in the departure of iodide ions and the steric rotation of the index salt group, which caused fluorescence quenching. The titration experiments showed that the probe had rapid qualitative and quantitative analysis capabilities for CN^−^. Moreover, the relevant biocompatibility experiments also demonstrated the potential application value of the probe.

## 1. Introduction

Cyanides are widely used in modern industries, such as gold and silver hydrometallurgy, electroplating technology, synthetic fibers, herbicides, etc. [1,2]. CN^−^ as an important anion that endangers human health, has received widespread attention. CN^−^ can interact with P450 in cytochrome, disrupting its ability to transport electrons in the respiratory chain, thereby damaging the central nervous system. Excessive CN^−^ can cause physiological disorders in the body, such as hypoxia, convulsions, dizziness, vomiting, vascular necrosis, and even death. According to a report by the World Health Organization (WHO), the CN^−^ concentration in drinking water is required to be below 1.9 μM [3,4,5]. With the increasing emphasis on physical health, it is necessary to develop a low-cost, simple-to-operate, and highly sensitive and selective CN^−^ detection method. At present, the common analytical methods for detecting CN^−^ mainly include high-performance liquid chromatography, mass spectrometry, atomic absorption spectroscopy, etc. [6,7,8]. However, there are drawbacks such as high testing costs and complex operations, which greatly limit their application scope. Organic fluorescent probes have advantages such as high selectivity and sensitivity, simple operation, and good biocompatibility, which can effectively eliminate the above-mentioned drawbacks [9,10,11].

So far, some CN^−^ probes have been reported, and their common sensing mechanisms mostly rely on strong hydrogen bonding interactions, coordination effects, Lewis acid-base binding effects, supramolecular self-assembly, and nucleophilic reactivity [12,13,14,15]. Among these, the electron-rich nucleophilic-attack-based CN^−^ fluorescence probe has high selectivity and sensitivity due to the specificity of the reaction; so, this type of probe is receiving more and more attention [16,17]. Generally speaking, the nucleophilic addition reaction mainly occurs between CN^−^ and activated carbonyl groups, α, β- Unsaturated carbonyl groups, dicyano vinyl groups, indole type groups, imine groups, or boron-containing compounds [18,19]. Therefore, based on this nucleophilic addition reaction mechanism, we can design fluorescent molecules containing the functional groups mentioned above that can specifically react with CN^−^ to prepare fluorescent probes. Indole, as a common fluorescent group, is undoubtedly a good choice. Indole compounds are a large class of compounds containing benzo five-membered nitrogen heterocyclic structural units, which are fused heterocyclic compounds formed by the benzene ring and pyrrole ring. They are also known as benzopyrrole and have good biological activity and environmental friendliness. As a chemical raw material, indole and its derivatives have a wide range of applications in various fields, such as the production of pharmaceuticals, pesticides, spices, dyes, feed, and food additives [20,21,22].

In this research, a novel indole-based CN^−^ probe was designed and prepared; it comprised an indole fluorophore as a CN^−^-specific reactive unit. The nucleophilic attack of CN^−^ on the carbon–nitrogen double bond in the probe inhibited the fluorescence release of indole fluorophore and resulted in fluorescence quenching. This probe not only possesses good selectivity and sensitivity but also has a fast response time, thus providing more practical applications than other types of probes.

## 2. Materials and Methods

### 2.1. Materials

All the chemicals and solvents in the experiments were purchased from commercial suppliers and used without further purification. Solutions of different anions (CN^−^, SO_4_^2−^, F^−^, OH^−^, Cl^−^, I^−^, H_2_PO_4_^−^, NO_3_^−^, HPO_4_^2−^, C_2_O_4_^2−^, BrO_3_^−^) in experiments were dissolved in HEPES-NaOH buffer solution at pH 7.4. The Probe 1 solution was prepared in N,N-Dimethylformamide (DMF). The test samples were prepared by adding the required concentration and volume of anionic solutions into accurate amounts of Probe 1 in DMF solutions. In fluorescence titration experiments, the excitation wavelength was set as 305 nm, and both the excitation and emission slit widths were set as 2.5 nm.

### 2.2. Methods

Probe 1 was characterized by ^1^H NMR and HRMS spectra (Figures S1 and S2). ^1^H NMR spectra were performed on a Varian mercury-300 spectrometer at an operating frequency of 300 MHz with TMS as an internal standard and DMSO-*d_6_* as solvent. High-resolution mass spectra (HRMS) were performed on Agilent 1290- micro TOF QII. The UV–Vis absorption spectra were taken on Shimadzu UV-2600. The fluorescence spectra measurements were taken on the Hitachi F-4500 spectrofluorimeter. The pH measurements were taken on Mettler–Toledo Instruments DELTE 320 pH. The cell imaging experiments employed HepG2 cells. The live cells were first incubated with Probe 1 solution (30 μM) for 0.5 h at 37 °C in a 5% CO_2_ atmosphere and washed 3 times with phosphate-buffered saline (PBS, pH = 7.4); then, CN^−^ solution (30 μM) was added into the Probe-1-merged cells for 30 min. HepG2 cells were adopted in the fluorescence cell image experiments, and the experimental equipment consisted of an Olympus IX-70 fluorescence microscope and a Olympus c-5050 digital camera.

### 2.3. Synthesis

**Scheme 1:**
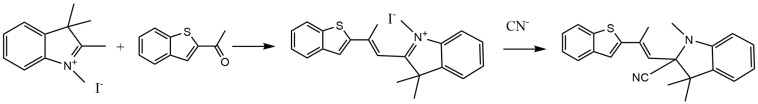
The synthesis process of Probe 1 and its reaction with CN^−^.

As illustrated in Scheme 1, Probe 1 was prepared through a simple one-step synthesis; the benzothiophene group and iodine compounds were connected by a Knoevenagel–Doebner condensation reaction containing an indolium group as a CN^−^ acceptor. 1,2,3,3-tetramethylindolyonium iodide (0.099 g, 0.33 mmol) and 2-acetyl- benzothiophene (0.058 g, 0.33 mmol) were dissolved in 50 mL anhydrous ethanol, stirred and mixed, and sonicated for 15 min. The mixture was refluxed under nitrogen protection for 18 h. After the reaction was completed, the reaction solvent was evaporated under reduced pressure and placed in a vacuum drying oven for 24 h to obtain a reddish brown solid. The crude product was separated and purified using column chromatography (developing agent:petroleum ether: ethyl acetate = 2:1) to obtain a reddish brown oily product. After rotary evaporation, a final yellow brown product of 0.117 g was obtained with a yield of 73.1%. ^1^H NMR (300 MHz DMSO, 25 °C, TMS): δ 0.72(s, 3H), 1.47(s, 6H), 2.31(s, 3H), 5.40(s, 1H), 7.23(m, *J* = 3.0, *J* = 3.0, 1H), 7.26(m, *J* = 6.0, *J* = 3.0, 1H), 7.27(m, *J* = 3.0, *J* = 6.0,1H), 7.62(m, *J* = 3.0, *J* = 6.0, 1H), 7.64(m, *J* = 3.0, *J* = 3.0, 1H), 7.66(m, *J* = 6.0, *J* = 3.0, 1H), 7.69(m, *J* = 3.0, *J* = 3.0, 1H), 8.16(d, *J* = 3.0, 1H), 8.25(d, *J* = 3.0, 1H). ^13^C NMR (75 MHz DMSO, 25 °C, TMS): 17.93, 25.02, 33.17, 56.12, 110.02,110.98, 112.48, 119.95, 122.11, 124.59, 125.95, 127.23, 129.92, 136.33, 139.19, 140.33, 142.51, 144.21, 172.13. ESI-MS m/z [M]^+^ calc. 459.05, obs. 459.4. (Figures S1–S3).

## 3. Results

### 3.1. UV Absorption Spectroscopy Testing

**Figure 1:**
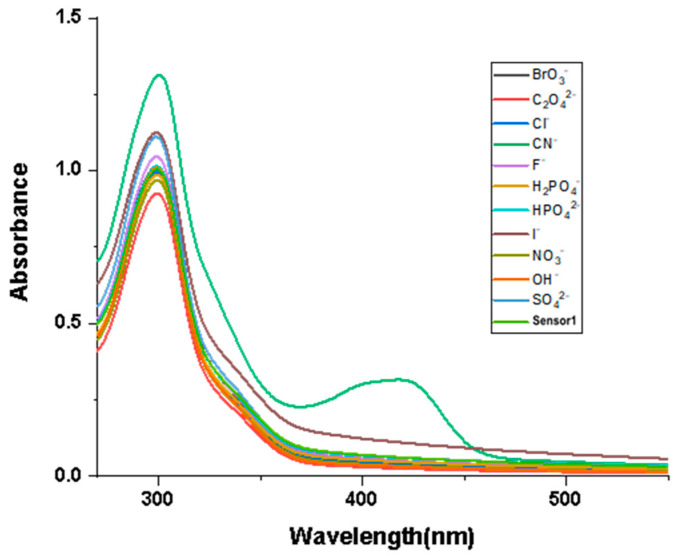
Absorption spectral response of Probe 1 to different anions [v(DMF)/v(H_2_O) = 1:1, pH = 7.4].

The influence of different anions on Probe 1 in the UV–Vis absorption spectrum was measured using titration experiments. The test conditions were in DMF and HEPES-NaOH buffer solutions [v(DMF)/v(H_2_O) = 1:1]. The concentration of Probe 1 and the anions (CN^−^, SO_4_^2−^, F^−^, OH^−^, Cl^−^, I^−^, H_2_PO_4_^−^, NO_3_^−^, HPO_4_^2−^, C_2_O_4_^2−^, BrO_3_^−^) in the test was 5 × 10^−4^ mol/L. Then, the same volume of anionic solution was added to the solution of Probe 1 for UV absorption spectroscopy measurement. As illustrated in Figure 1, among the tested anions, only CN^−^ affected the UV–Vis absorption spectrum of Probe 1, with a new absorption peak appearing at 430 nm, while the other anions did not have an impact. In addition, the maximum absorption peak was observed at 305 nm; therefore, 305 nm was used as the excitation wavelength for subsequent fluorescence spectroscopy measurements.

### 3.2. Fluorescence Emission Spectroscopy Testing

**Figure 2:**
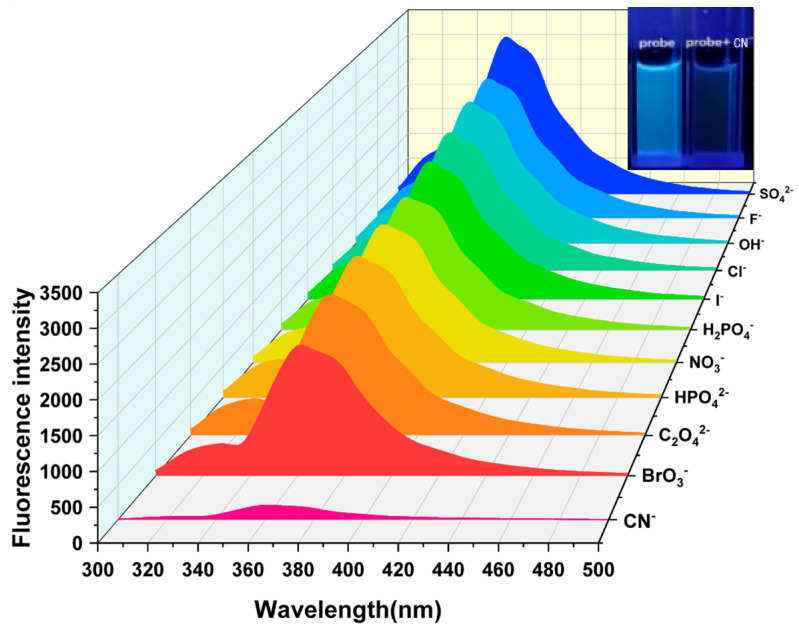
Fluorescence emission spectral response of Probe 1 to different anions [v(DMF)/v(H_2_O) = 1:1, pH = 7.4].

**Figure 3:**
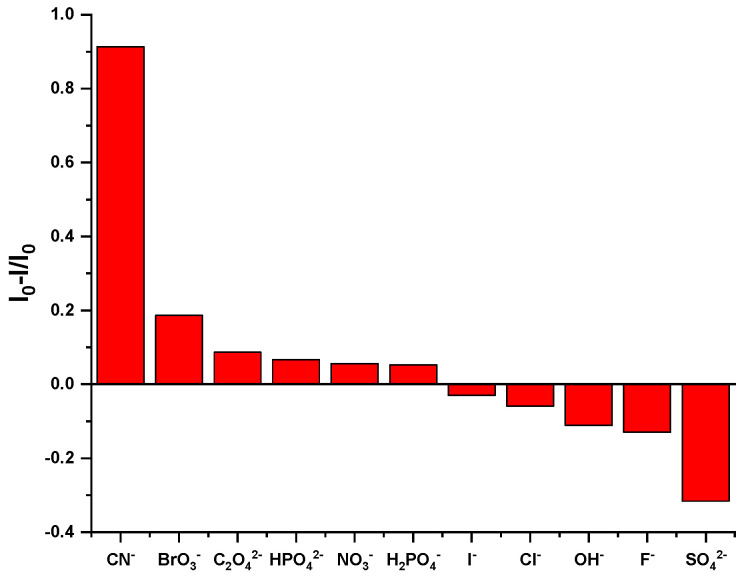
Fluorescence quenching degree of different anions in Probe 1 at 361 nm in the fluorescence emission spectrum.

Selective experiments are an important method for evaluating the recognition ability of fluorescent probes. By detecting the changes in fluorescence signals after different substances are added to the probe, the specificity of the probe in recognizing the analyte can be effectively demonstrated. To detect the influence of common anions on the fluorescence intensity of Probe 1, a series of anions were used in selective experiments. Under the same experimental conditions, equal volumes of 5 × 10^−4^ M HEPES NaOH buffer solutions containing different kinds of anions were added to the DMF solution of 5 × 10^−4^ M Probe 1 to prepare experimental samples. As illustrated in Figure 2, Probe 1 exhibited an emission peak at 361 nm. The introduction of SO_4_^2−^, F^−^, OH^−^, Cl^−^, I^−^, H_2_PO_4_^−^, NO_3_^−^, HPO_4_^2−^, C_2_O_4_^2−^, and BrO_3_^−^ had no obvious effect on fluorescence emission, but CN^−^ significantly weakened the fluorescence intensity at 361 nm. In addition, the quenching degree of the Probe 1 solution on different anions was also measured. As shown in Figure 3, only CN^−^ had a significant quenching degree on Probe 1, reaching about 91%, while the other anions did not have this significant effect.

**Figure 4:**
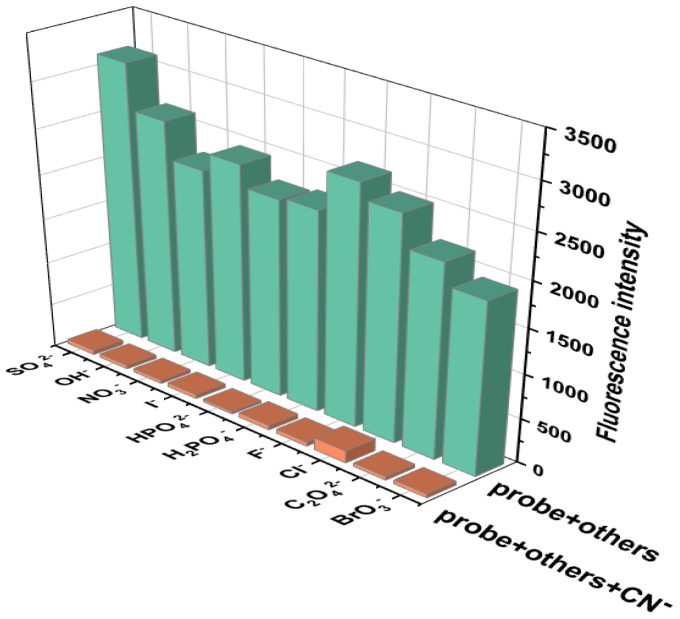
Competitive experiment of Probe 1 (5 × 10^−4^ M) for detecting CN^−^ in the presence of interfering anions (5 × 10^−3^ M), [v(DMF)/v(H_2_O) = 1:1, pH = 7.4, λ_ex_ = 305 nm, λ_em_ = 361 nm, slits: 2.5 nm/2.5 nm].

The anti-interference ability of Probe 1 in CN^−^ recognition was further verified through competitive experiments. The competitive experiment is a further extension of the selective experiment, which involves adding a large amount of interfering substances to the probe–analyte system to determine whether it will interfere with the detection signal. Firstly, 1.5 equivalents of CN^−^ solution was added to the DMF solution of Probe 1 to induce fluorescence quenching. Afterwards, 10 equivalents of SO_4_^2−^, F^−^, OH^−^, Cl^−^, I^−^, H_2_PO_4_^−^, NO_3_^−^, HPO_4_^2−^, and C_2_O_4_^2−^ were added to the Probe 1-CN^−^ solution separately, and the emission spectrum on a fluorescence spectrometer was recorded under the excitation wavelength of 305 nm. The fluorescence intensities of the emission spectra of the mixed systems at 361 nm were used as the competitive experimental data. And the selective experiments were adapted to compare with the competitive experiments. As shown in Figure 4, the green bar chart represents the selective experiments, and the orange bar chart represents the competitive experiments. From the orange bar chart, it can be seen that even in the presence of 10 equivalents of interfering anions, Probe 1 could still produce a targeted fluorescence intensity response to CN^−^ under complex conditions, which also proves that Probe 1 had good selectivity and anti-interference ability. This good recognition ability can serve as a basis for the quantitative analysis of cyanide ions. Therefore, we believe that Probe 1’s selectivity to CN^−^ is more stable than other anions, which proves its ability to detect CN^−^ in complex systems.

**Figure 5:**
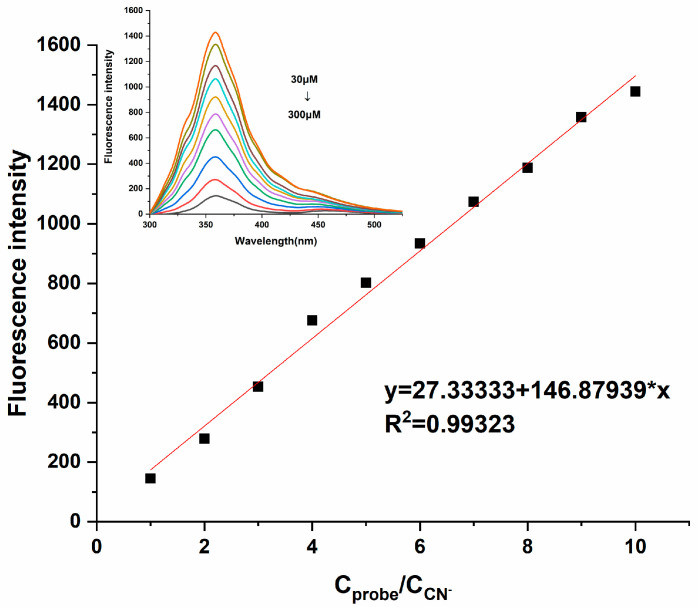
Fluorescence emission response of Probe 1 (300 μM) in different concentrations of CN^−^ (30−300 μM) [v(DMF)/v(H_2_O) = 1:1, pH = 7.4, λ_ex_ = 350 nm, slits: 2.5 nm/2.5 nm].

According to a report by the World Health Organization (WHO), the CN^−^ concentration in drinking water is required to be below 1.9 μM. Therefore, whether the probe can detect CN^−^ at low concentrations is particularly important, which also reflects the practical application value of Probe 1. Therefore, sensitivity experiments were naturally introduced into this research; the fluorescence signal changes for Probe 1 (3 × 10^−4^ M) in different concentrations of CN^−^ (3 × 10^−5^ M~3 × 10^−4^ M) were investigated in DMF and HEPES-NaOH buffer solutions [v(DMF)/v(H_2_O) = 1:1]. As illustrated in Figure 5, the fluorescence intensity of Probe 1 at 361 nm decreased gradually with the increasing concentrations of CN^−^ (0.1–1 equivalent). Linear fitting was performed based on the fluorescence intensity attenuation at 361 nm, and it was found that this fluorescence attenuation was linearly correlated. The linear equation y= 146.87939x + 27.33333 was obtained, with R^2^ = 0.99323. From this, it could be concluded that the probe could effectively quantitatively analyze CN^−^. The detection limit (*DL*) of Probe 1 to CN^−^ was calculated from the following equation [23]:

(1) $$DL=\frac{K\times Sb_{1}}{S}$$

In the equation, *K* is the constant 2, *Sb_1_* is the standard deviation of the blank solution, and *S* is the slope of the calibration curve. After calculation, the detection limit of Probe 1 to CN^−^ was 1.53 × 10^−6^ M, and it was below the concentration specified by the World Health Organization.

**Figure 6:**
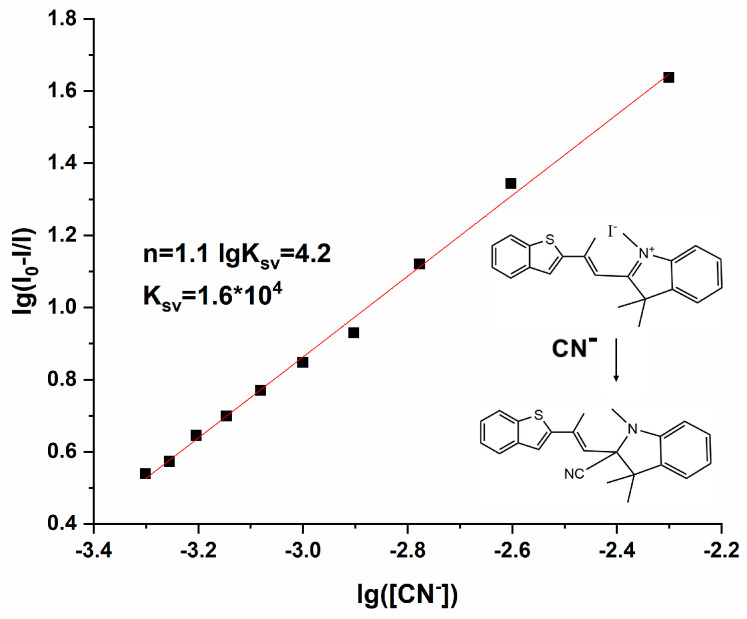
The linear fitting of the fluorescence titration curve of Probe 1-CN^−^ [v(DMF)/v(H_2_O) = 1:1, pH = 7.4, λ_ex_ = 350 nm, slits: 2.5 nm/2.5 nm].

To further verify the detection ability of Probe 1 for CN^−^, the binding constant and binding ratio of Probe 1 to CN^−^ were determined from the Stern–Volmer equation [24], As illustrated in Figure 6, the concentration of CN^−^ was diluted gradually from 5 × 10^−3^ M to 5 × 10^−4^ M, and the corresponding data were substituted into Equation (2):

(2) $$\mathrm{lg}(\frac{I_{0}-I}{I})=\mathrm{lg}K_{SV}+\mathrm{nlg}(Q)$$

wherein *I*_0_ is the fluorescence intensity of Probe 1, *I* is the fluorescence intensity of Probe 1-CN^−^, *K_SV_* is the Stern–Volmer constant, *n* is the number of binding sites, and *Q* is the concentration of CN^−^. By using the slope and intercept of the linear equation of the Stern–Volmer equation in Figure 6, we calculated that *K_SV_* is 1.6 × 10^4^ M^−1^, and *n* equals 1.1. So, the complexation constant of Probe 1 with CN^−^ was 1.6 × 10^4^ M^−1^, and the binding ratio was 1:1, verifying once again the nucleophilic attack reaction of CN^−^ on Probe 1, and the reaction mechanism is shown in Figure 6. The introduction of CN^−^ leads to stable covalent bridging between CN^−^ and the strongly electrophilic quaternary ammonium salt N atom, causing a change in the spatial electron arrangement of the N atom, resulting in a spatial twist of the entire indole group and a change in the angle between its spatial plane and the benzothiophene group, forming a new spatial structure accompanied by changes in the fluorescence emission [25,26]. Compared with other reported CN^−^ probes, the electron-rich nucleophilic-attacking CN^−^ fluorescent probe provides good selectivity and sensitivity and avoids interference from F^−^ in the hydrogen-bonding CN^−^ probe. Moreover, the synthesis process is relatively simple and convenient and accompanied by significant spectral changes, so Probe 1 is provided with potential applicability on account of the above advantages.

**Figure 7:**
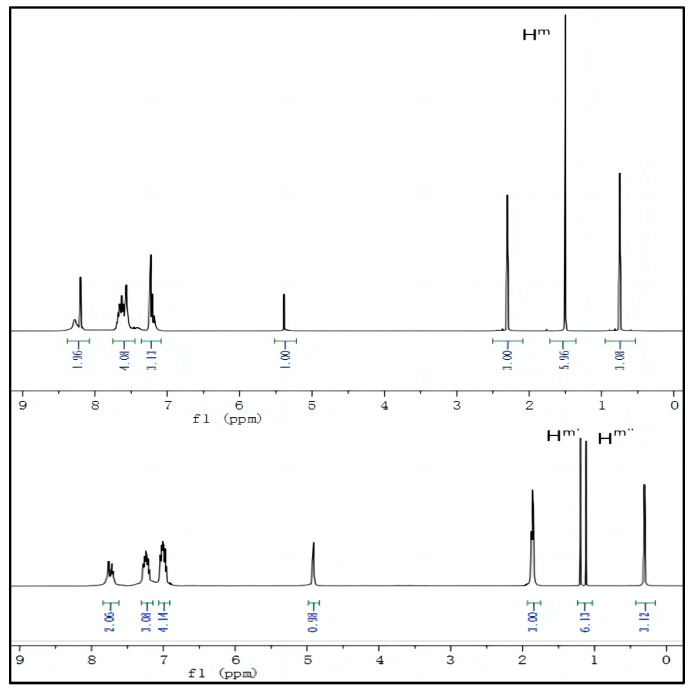
^1^H NMR spectra of Probe 1 and Probe 1 -CN^−^.

To further investigate the mechanism of Probe 1 recognizing CN^−^, we compared ^1^H NMR of Probe 1 before and after adding CN^−^ to analyze the attacking position of CN^−^. As shown in Figure 7, after adding CN^−^, all spectral positions shift towards higher fields, while the original two methyl nuclear magnetic positions on the indoleum salt group are uniformly labeled as H^m^ because there is no difference. After the CN^−^ nucleophilic attack, due to changes in spatial structure, it splits into H^m^′and H^m^′′, and the peak position of the hydrogen atom on the methyl group connected to the quaternary ammonium salt shifts to a higher field by about 1 ppm. From this phenomenon, the following conclusions can be drawn: (1) CN^−^ nucleophilic attack on electron-deficient carbon atoms causes a decrease in the electron-withdrawing ability of the indoleum iodide group, resulting in all hydrogen spectra shifting to a higher field; (2) The two methyl groups become nonequivalent after the addition of CN^−^, resulting in splitting into two peaks. This conclusion validates the initial experimental hypothesis.

**Figure 8:**
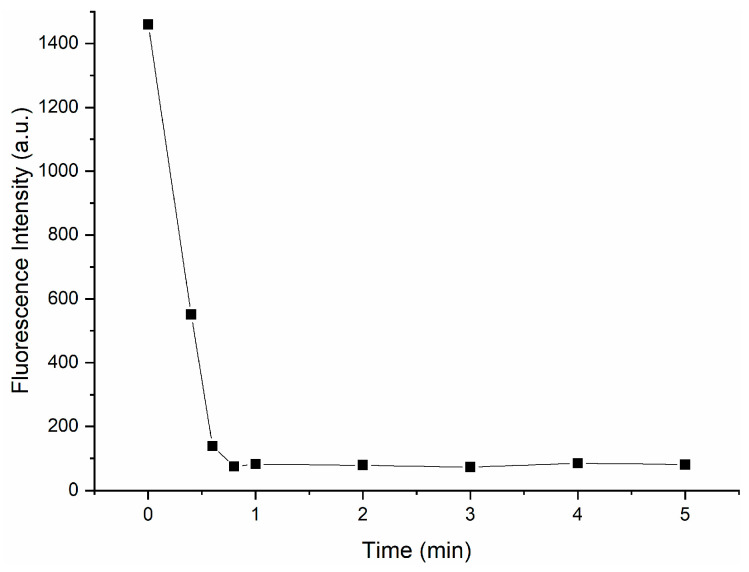
Response time of Probe 1 (300 μM) upon addition of CN^−^(300 μM).

Response time is an important factor in measuring the detection capability and practical application value of probes. Therefore, we studied the response time of Probe 1 to CN^−^. As shown in Figure 8, experimental results illustrate that fluorescence quenching was completed within one minute when CN^−^ was added to the solution; so, Probe 1 could serve as a rapid CN^−^ monitor.

### 3.3. Biocompatibility Experiments

**Figure 9:**
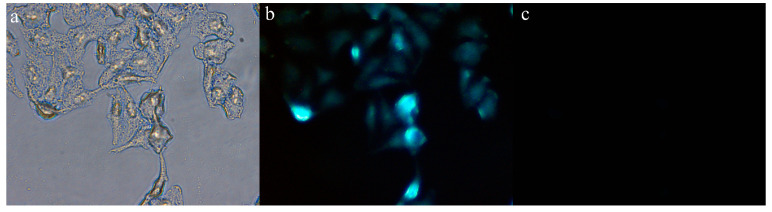
Cell fluorescence imaging experiment of Probe 1: (**a**) Bright field image of HepG2 cells incubated with Probe 1; (**b**) HepG2 cells incubated with Probe 1; (**c**) HepG2 cells incubated with Probe 1-CN^−^.

To further explore the biological application of Probe 1, cell imaging experiments were used to determine whether Probe 1 could track intracellular CN^−^. The experiments were conducted in HepG2 cells at 37 °C in a 5% CO_2_ atmosphere. The live HepG2 cells were first incubated with Probe 1 solution (30 μM) for 0.5 h and washed 3 times with phosphate-buffered saline (PBS). Then, CN^−^ solution (30 μM) was added into the Probe-1-merged cells. And the cell images were collected after 30 min. As illustrated in Figure 9a,b, the cells treated with Probe 1 displayed clear contours and fluorescence emission, yet the cells that had been treated with CN^−^, as shown in Figure 9c, exhibited a significant fluorescence quenching effect. Therefore, based on the above fluorescence imaging experiments, we believe that Probe 1 can be effectively applied to the detection of CN^−^ in cells.

**Figure 10:**
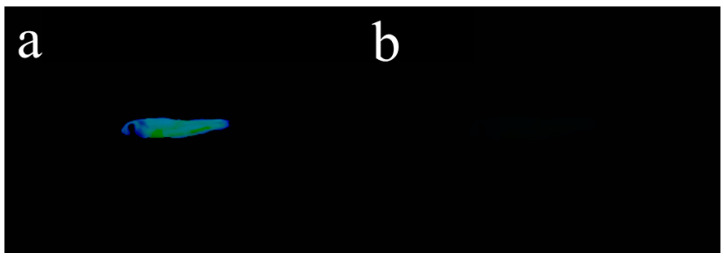
(**a**) Zebrafish bred with only Probe 1 under UV light; (**b**) Zebrafish bred with Probe-1-CN^−^ under UV light.

In addition to exploring the fluorescence tracing application of Probe 1 in cells, we also introduced Probe 1 into live zebrafish experiments to verify its biocompatibility. Zebrafish generally have a lifespan of 2–3 years, with a maximum of 5.5 years. Due to its ease of breeding, short reproductive period, and large egg production, it has become an important research target in the field of biological science. The zebrafish were fed with 10 μM Probe 1 in DMSO for 20 min and washed 3 times with PBS buffer solution. Then, 10 μM CN^−^ solution was added into Probe-1-treated zebrafish. After 1 h, the fluorescence images of zebrafish were collected under 365 nm ultraviolet light. As shown in Figure 10a, the fish treated with Probe 1 emitted obvious fluorescence under UV-light; however, as shown in Figure 10b, the subsequent addition of CN^−^ would quench the fluorescence triggered by Probe 1. From this, the above biocompatibility experiments revealed that Probe 1 was able to identify CN^−^ in cells and can also be used for CN^−^ detection in live zebrafish, with broad application prospects.

## 4. Conclusions

In this research, a novel indolium-based fluorescent probe for recognizing CN^−^ was synthesized. The addition of CN^−^ in Probe 1 caused remarkable fluorescence quenching due to the nucleophilic addition reaction between CN^−^ and C = N, which suppressed fluorescence release from Probe 1. The titration experiments verified that Probe 1 has good selectivity and sensitivity towards CN^−^ and could effectively perform qualitative and quantitative analysis on CN^−^, and after calculation, it was found the detection limit could reach 1.53 × 10^−6^ M. According to the Stern–Volmer equation, the binding constant of Probe 1 to CN^−^ was calculated to be 1.6 × 10^4^ M^−1^ and the binding model between Probe 1 and CN^−^ was also validated. In terms of biological applications, both cell imaging experiments and live zebrafish experiments have demonstrated that Probe 1 has good biocompatibility and is able to effectively detect CN^−^ in living organisms.

## Data Availability

The data presented in this study are available in Supplementary Material.

## Supplementary Materials

The following supporting information can be downloaded at: https://www.mdpi.com/article/10.3390/bios14050244/s1, Figure S1: ^1^H NMR spectrum of Probe 1; Figure S2: ^13^C NMR spectrum of Probe 1. Figure S3: LC-MS of Probe 1.
