# Supplementary material for: A Novel Indolium-Based Fluorescent Probe for Fast Detection of Cyanide
Source: Biosensors (Basel). 2024 May 13;14(5):244. doi: 10.3390/bios14050244 (PMC11118950; doi:10.3390/bios14050244)
Supplement: Supplementary file 1 [file biosensors-14-00244-s001.zip › biosensors-2989023-supplementary.pdf]

A Novel Indolium-based Fluorescent Probe for Fast Detection of Cyanide

Mei Ding[1], Xiao Xiao[1], Chen Zhou [1]*, Mingxin Luo[1] and Jing Sun[1]

[1] School of Chemistry & Environmental Engineering, Jilin Provincial International Joint Research Center of Photo-functional Materials and Chemistry, Changchun University of Science and Technology, Changchun, 130022, People's Republic of China
Corresponding author: Chen Zhou
E-mail address: zhouchen@cust.edu.cn
Tel: +86431 85583894

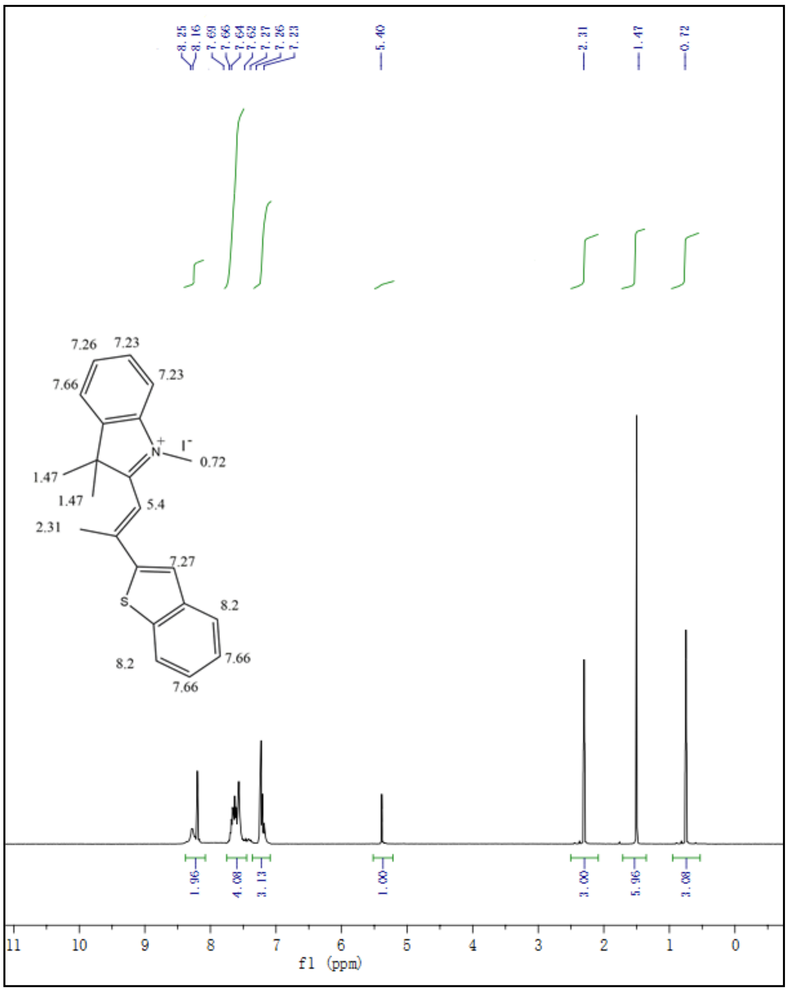

Figure S1. ^{1}H NMR spectrum of Probe 1

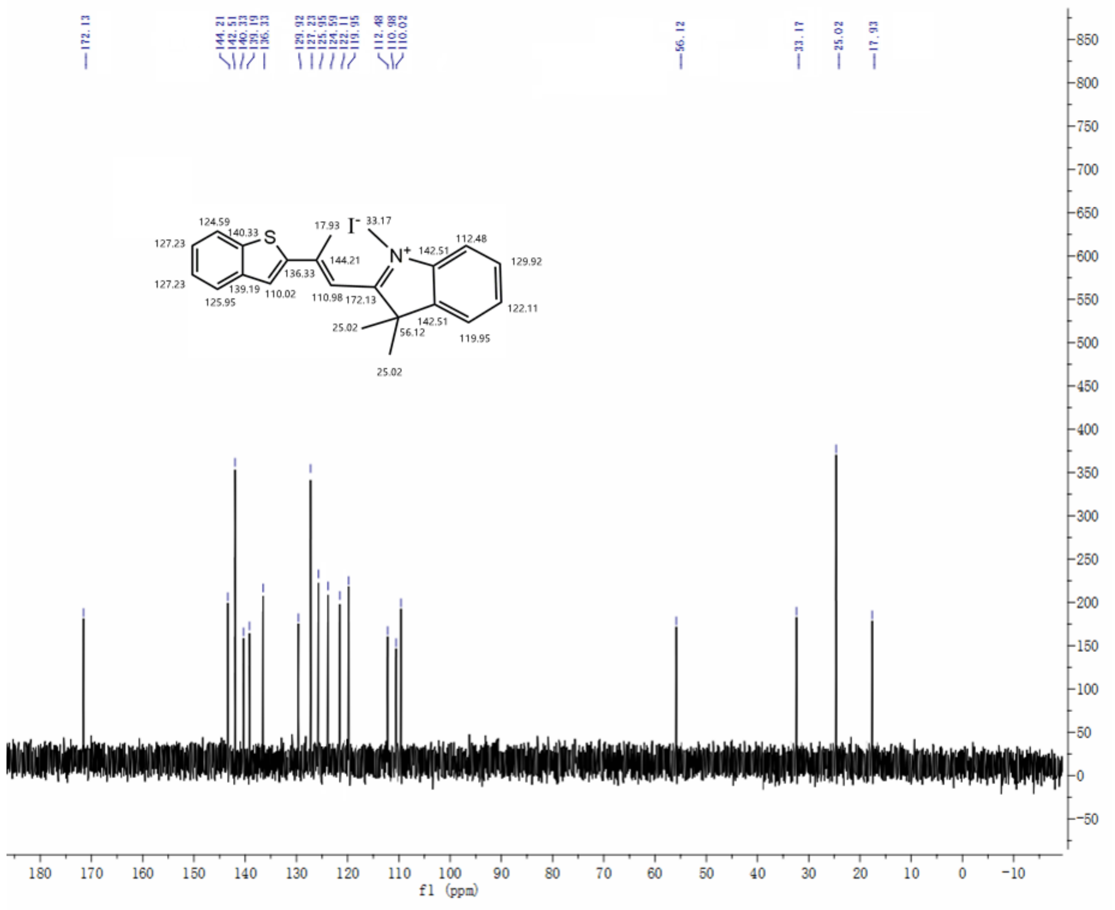

Figure S2. ^{13}C NMR spectrum of Probe 1

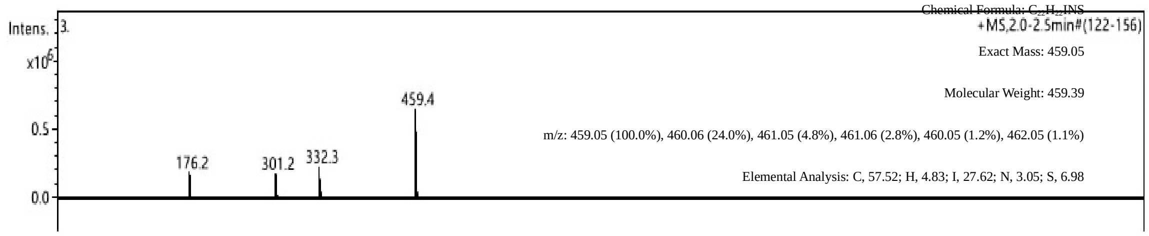

Figure S3. LC-MS of Probe 1
